# Differences and mechanisms underpinning a change in the knee flexion moment while running in stability and neutral footwear among young females

**DOI:** 10.1186/s13047-018-0307-9

**Published:** 2019-01-08

**Authors:** Timothy A. Sayer, Rana S. Hinman, Kade L. Paterson, Kim L. Bennell, Karine Fortin, J. Kasza, Adam L. Bryant

**Affiliations:** 1 Centre for Health Exercise & Sports Medicine, Department of Physiotherapy, The University of Melbourne, Alan Gilbert Building (Level 7), 161 Barry St, Parkville, Melbourne, 3052 Australia; 2 LUNEX International University of Health, Exercise and Sports, Differdange, Grand Duchy of Luxembourg; 3 Department of Epidemiology and Preventive Medicine, Monash University, Clayton, VIC Australia

**Keywords:** Kinetics, Female, Adolescent, Footwear

## Abstract

**Background:**

Higher peak external knee flexion moments (KFM) during running has been observed in healthy people wearing athletic footwear compared to barefoot, which may increase risk of knee pathologies such as patellofemoral pain. Currently, no studies have examined whether stability and neutral style athletic shoes influence the peak KFM differently, or explored the underlying biomechanical mechanisms by which footwear alters peak KFM in young females.

**Methods:**

Lower limb biomechanics of sixty girls aged between 10 and 25 years old were collected while running in footwear (both stability and neutral) and barefoot. The external peak KFM, sagittal plane kinematics, sagittal plane knee ground reaction force (GRF) lever arm and sagittal plane resultant GRF magnitude were analysed by repeated measures Analysis of Variance. Linear mixed models were fit to identify predictors of a change in peak KFM, and to determine if the effects of these predictors differed between footwear conditions.

**Results:**

The peak KFM was higher wearing both shoe styles compared to barefoot (*p* < 0.001), while no between-shoe differences were found (*p* > 0.05). Both shoes also increased kinematic variables at the hip, knee, and ankle (*p* < 0.05)*.* When all these variables were entered into the mixed model, only a change in the knee-GRF lever arm was predictive of a change in peak KFM wearing shoes compared to barefoot (*p* < 0.001).

**Conclusion:**

These findings provide evidence that stability and neutral shoes increase peak KFM compared to barefoot, which is associated with a change in the knee-GRF lever arm rather than a change in lower limb kinematics. Future studies may consider manipulating footwear characteristics to reduce the knee-GRF lever arm in an effort to reduce peak KFM and the potential risk of patellofemoral pain.

**Electronic supplementary material:**

The online version of this article (10.1186/s13047-018-0307-9) contains supplementary material, which is available to authorized users.

## Introduction

Running is a popular exercise associated with a healthy lifestyle. Despite its benefits however, the repetitive nature of running can lead to musculoskeletal injuries [1], with patellofemoral pain (PFP) being one of the most common [2]. Specifically, a high incidence of PFP is reported amongst adolescent females, with 15–30% developing the condition [3, 4]_._ and many experiencing recurrent symptoms into adulthood [5].

Although the causes of PFP amongst adolescent females are multifactorial [5–7], altered sagittal plane knee biomechanics such as a higher peak knee flexion moment (KFM) may be a contributor [8, 9]. For instance, higher peak KFM is associated with higher patellofemoral joint loads which, in turn, can increase risk of developing PFP [8, 9]. Higher peak KFMs may be driven by growth-related factors associated with female pubertal development [10–12]. Indeed, a recent study published by our group confirmed that girls classified as early/mid- and late/post-pubertal development exhibit higher barefoot running-related peak KFM compared to their pre-pubertal counterparts [13]. Given that the girls in the aforementioned study are also at higher risk of developing knee pathologies such as PFP [3, 14–16], further studies should consider the biomechanical mechanisms contributing to higher peak KFM in this cohort.

Girls and young adults typically wear a variety of athletic footwear when running. On the basis of previously published criteria [17], athletic shoes are usually classified as ‘stability’ or ‘neutral’ shoes. Stability shoes typically possess increased medial, midfoot and longitudinal stiffness and support, whilst these characteristics are typically lower or absent in neutral shoes [17]. Combined, these shoe features have been shown to modify foot and knee frontal plane mechanics [18, 19]. In addition, other footwear features such as pitch (i.e., heel to toe offset) and midsole thickness, typically higher in stability compared to neutral footwear, likely influence sagittal plane knee moments [8, 20, 21]. Mechanistically, greater footwear pitch reduces peak ankle dorsiflexion angle and increases peak knee flexion angle [22, 23], while increased midsole thickness appears to increase knee flexion excursion compared to barefoot [21]. Hence, it is plausible that these footwear-related kinematic changes contribute to an elevation in running-related peak KFM [8, 20]. Whilst no previous studies have investigated whether peak KFM differs between stability and neutral footwear and barefoot in young females, such an investigation is important to clarify which type of footwear is likely to be most effective at reducing the risk of developing PFP [3, 4].

Although the available literature suggests footwear increases peak KFM [8, 20], the biomechanical mechanisms contributing to this phenomenon remain unknown. To date, no published studies have investigated the underlying mechanisms by which stability and neutral shoes may alter running-related peak KFM. Understanding mechanisms may help guide footwear manufacturers and researchers about more optimal footwear designs to lower injury risk. As discussed, stability and neutral footwear may increase, to a lesser or greater extent, the peak KFM by augmenting a change in lower limb kinematics, via decreased dorsiflexion and increased knee flexion angles compared to barefoot [20, 22, 23]. Higher peak knee flexion wearing footwear likely leads to an increased perpendicular distance (mm) from the knee joint centre (KJC) to GRF vector (i.e., the knee-GRF lever arm), resulting in higher peak KFM; however, this notion is yet to be confirmed. Likewise, the sagittal plane resultant GRF magnitude may also be influenced by footwear [24], and thus may be another potential contributor to alterations in the peak KFM. Hence, lower limb kinematics, the knee-GRF lever arm and sagittal plane resultant GRF magnitude are likely relevant variables to explore as potential mechanisms underpinning changes in peak KFM observed with footwear during running.

The primary aim of this study was to examine differences in running-related peak KFM between barefoot, stability and neutral footwear in adolescent girls and young women spanning early to post-puberty. A secondary aim was to determine whether the knee-GRF lever arm, sagittal plane resultant GRF magnitude and sagittal-plane kinematics (i.e., hip flexion angle, knee flexion angle, ankle dorsiflexion angle all at time of peak KFM, knee flexion excursion and knee flexion angle at initial contact) were associated with the change in peak KFM between footwear conditions. The primary hypothesis was that both the stability and neutral shoes would increase peak KFM compared to barefoot, but that the increase in peak KFM would be less with the neutral shoes.

## Methods

### Participants

This was a nested cohort study based on a previous related study in which higher peak KFM was found during running in both early/mid- and late/post-pubertal groups compared to pre-pubertal girls; however, no differences between early/mid- and late/post-pubertal groups were observed [13]. Moreover, no between group differences were reported for the peak knee abduction moment or knee internal rotation moment. Thus, the present study included the 60 early/mid- and late/post-pubertal girls, which is relevant in context of PFP given that this population is generally at higher risk of the condition compared to pre-pubertal girls [3, 4].

A detailed description of study participants and pubertal classification can be found in our previous study [13]. Briefly, girls were recruited from local sporting clubs surrounding the University of Melbourne Parkville campus. All participants included were healthy, physically active girls with a healthy weight (i.e., body mass index < 30 kg/m^2^). Girls were excluded if they: (i) had a history of lower limb injury, knee pain or medical condition that currently affected walking, running or jumping, (ii) previous anterior cruciate ligament, meniscal or PFJ injury, (iii) use of a bi- or tri-phasic oral contraceptive pill (OCP), (iv) any medically prescribed or over the counter orthotic worn in the past 6 months and (v) unable to speak write or read English. Written informed consent was obtained from the participant or her parent/guardian with prior ethics approval from the University of Melbourne Human Ethics Advisory Group (HEAG) and Human Ethics Sub Committee (HESC; application ID#: 1442604).

Information regarding menarche informed the appropriate time for biomechanical testing [13] as fluctuating estradiol levels in girls during and post-puberty may influence lower limb biomechanics [25, 26]. Participants who indicated that they had experienced menarche, but were not using a monophasic OCP, were tested within the first 7 days of their menstrual cycle (i.e., early follicular phase). In contrast, girls who had not experienced menarche or were using a monophasic OCP were tested anytime. To confirm that eumenorrheic participants were tested at the time of low estradiol levels (i.e., < 18 pmol/L according to the reference ranges for the follicular phase), a 5 mL saliva sample was provided immediately before biomechanical testing. Samples were sent to the manufacturer (Nutripath Integrative Pathology, Melbourne, Australia) for analysis via enzyme immunoassay.

**Fig. 1:**
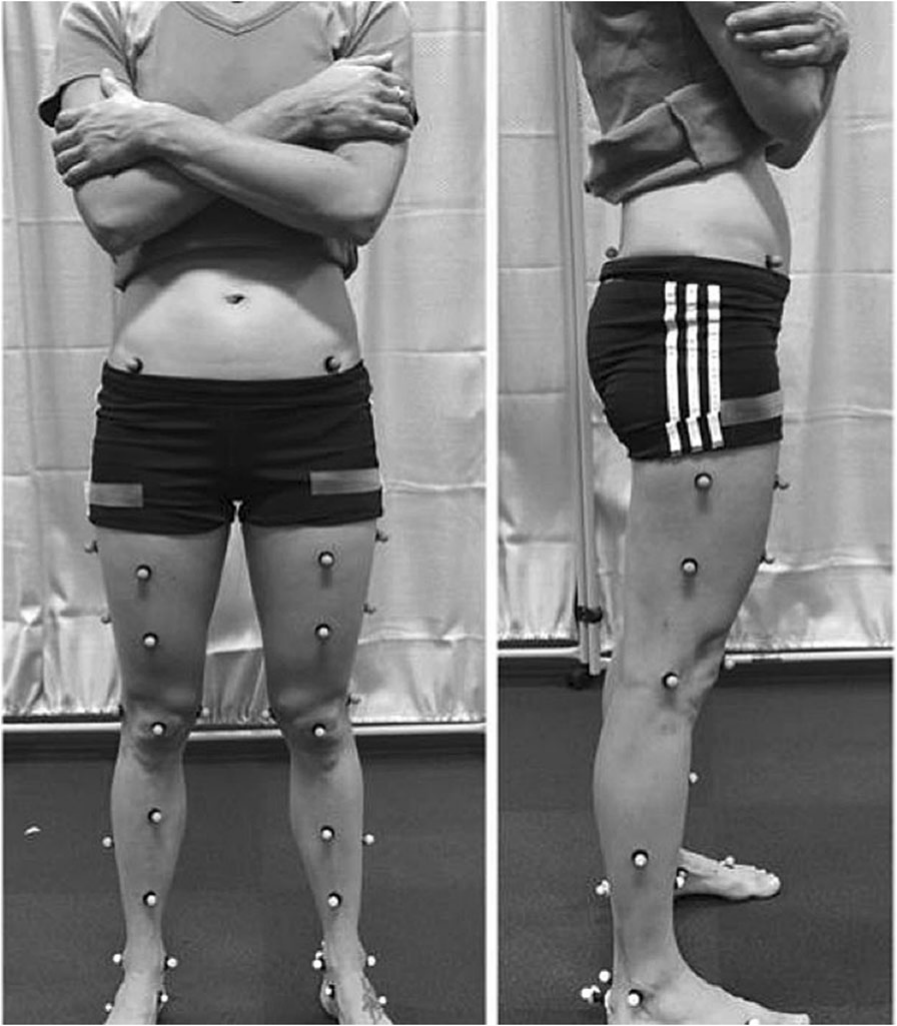
Participant prepared for data collection. All 40 (13 mm) reflective markers were placed on the trunk, thigh, shank and foot. Participants were instructed to fold their hands across their chest (as pictured) for the static calibration

Descriptive measures of height and weight were recorded barefoot. Limb dominance was then determined using the footedness subscale of the Lateral Preference Inventory (LPI) [27]. Following this, forty 13 mm retroreflective markers were adhered to each participant’s trunk, thigh, shank and foot according to a model previously described by Schache and Baker (Fig. 1) [28].

### Running task

The running task was described to participants using a standardized set of instructions that emphasized the importance of completing each trial using their natural running style [13]. No instructions/corrections were given about running technique. All participants were allowed approximately 5 minutes to familiarize themselves with running in each footwear condition in the laboratory. Following this, participants were required to complete three successful running trials in each condition: i) barefoot, ii) neutral shoes and, iii) stability shoes, with the order of the trials pre-determined via block randomization.

A successful trial involved (i) a clean strike of force plate with the dominant foot (i.e., foot contact was within all borders of the plate) and (ii) running speed 2.8–3.2 m/s (measured via photoelectric timing gates). Running speed was controlled given that variations between participants can influence joint kinematics and GRF and, in turn, net joint moments [29]. In the event a participant ran faster or slower than the designated time, instructions were given to adjust speed accordingly until the correct speed was attained via the photoelectric timing gates. A secondary analysis of running speed was then conducted post-session to confirm that each participant ran at the required velocity. Velocity was derived by calculating the average (m/s) across the three trials from the mid-point of the anterior superior iliac spine markers from the biomechanical model. If a participant did not run between the designated speed, they were excluded from the study.

### Footwear

There is currently no agreed definition for classification of shoes into stability and neutral shoes. Therefore, criteria were developed a priori based on features outlined in the Footwear Assessment Tool (FAT), including increased medial compared to lateral midsole density, and greater stiffness of the heel counter, midfoot and longitudinal aspects of the shoe [17]. Specifically, stability shoes were deemed to possess: (i) a midsole that was denser medially than laterally (i.e., medial post), (ii) < 10° midfoot frontal plane (torsional) stiffness, (iii) < 10° heel counter stiffness and (iv) < 45° midfoot sagittal plane (longitudinal) stability. Based on these criteria, the stability shoes tested in the present study had a score of 9 on the motion control properties scale of the FAT [17]. In contrast, the neutral shoes were deemed to possess: (i) a uniform midsole density, (ii) 10–45° heel counter stiffness (iii) 10–45° torsional stiffness and (iv) > 45° midfoot longitudinal stiffness. In combination, these features contribute to a score of 3 on the motion control properties scale of the FAT [17]. As a result, the Asics Kayano-GS was selected as the stability shoe and the Asics Zaraca 3 as the neutral shoe.

Further technical features related to the high support shoes (Asics Kayano-GS) include: (i) heel stack height = 25 mm, (ii) forefoot stack height = 12 mm, (iii) footwear pitch = 13 mm and (iv) shoe mass = 260 g. For the low support shoes (Asics Zaraca 3) these features included: (i) heel stack height = 28 mm, (ii) forefoot stack height = 18 mm, (iii) footwear pitch = 10 mm and (iv) shoe mass = 240 g (Additional file 1: Figure S1). Both shoes were the current model at time of testing.

### Motion analysis

Kinematic (120 Hz) and GRF data (2400 Hz) were collected using a 12-camera Vicon motion analysis system (Oxford, UK) synchronized to a concealed force plate (AMTI, Inc., Watertown, MA, USA). Data were filtered using a fourth order zero-lag Butterworth low-pass filter with a cut-off frequency of 20 Hz. As per Schache & Baker [28], the kinematic model included eight rigid body segments; trunk, pelvis, two thighs, two legs and two feet. Joint moments were calculated from the GRF and kinematic data for the hip, knee and ankle across the whole stance phase using inverse dynamics and expressed in the distal anatomical reference frame normalized to bodyweight (Nm/kg; [28]). All biomechanical variables tested in this study are outlined in Table 1. Positive kinematic and kinetic values indicate flexion whilst negative indicates extension. The sagittal plane knee-GRF lever arm and the resultant sagittal plane GRF magnitude were both derived from a custom-written Body Builder program (Vicon, Oxford, UK) and averaged across three running trials. Descriptive data pertaining to the anthropometric segment lengths for the thigh and shank were derived and extracted from the kinematic model.

**Table 1:** Biomechanical variables of interest between footwear and barefoot conditions

Variable	Definition
Peak KFM (Nm/kg)	Peak external knee flexion moment during stance. Positive values indicate higher KFM.
Stance time (s)	Time of stance from initial contact (> 20 N) until toe off (< 20 N).
Sagittal plane knee-GRF lever arm (mm)	Perpendicular distance between GRF and knee joint centre in laboratory sagittal plane. Calculated at time of peak KFM.
Sagittal plane resultant GRF magnitude (BW)	Resultant magnitude of the sagittal plane GRF calculated at time of peak KFM. Force was converted from Newton’s to bodyweight (BW).
Knee flexion angle (°)	Sagittal plane knee flexion angle at time of peak KFM. Positive values indicate knee flexion.
Ankle dorsiflexion angle (°)	Sagittal plane ankle dorsiflexion angle at time of peak KFM. Positive values indicate dorsiflexion.
Hip flexion angle (°)	Sagittal plane hip flexion angle at time of peak KFM. Positive values indicate hip flexion.
Knee flexion excursion (°)	Difference between the knee flexion angle at initial contact and peak across stance phase.
Knee flexion at initial contact (°)	Knee flexion angle at initial contact of force plate. Initial contact designated as time when GRF > 20 N.

*BW* bodyweight, *KFM* external knee flexion moment, *GRF* ground reaction force

### Statistics

Descriptive data (i.e., means and SD) were calculated for all outcome variables. Repeated measures ANOVA was used to examine differences between footwear conditions (barefoot, stability and neutral) for each biomechanical variable outlined in Table 1. In the event of a significant main effect of footwear condition, *post-hoc* analysis using Fisher’s Least Significant Difference tests were performed, whereby the mean difference (MD) and 95% confidence intervals (CI) were reported for all significant variables.

Following this, the difference between the baseline measurement and each of the stability shoe and neutral shoe measurements was taken and considered as the dependent variable in regressions. A linear mixed model with a random intercept for participant was fit to account for the potential similarity of measurements on each subject and identify variables predictive of the change in peak KFM (i.e., dependent variable) wearing shoes compared to barefoot. Before fitting this model for change in peak KFM, a preliminary step was performed to determine if any interactions between footwear condition and biomechanical predictors (Table 1) should be included in the final model (i.e., if the effect of any predictors of change in peak KFM from barefoot depended on the type of shoe worn, Additional file 2: Table S1). If an interaction between footwear condition and each of the change in lower limb kinematics, change in sagittal plane knee-GRF lever arm, change in sagittal plane resultant GRF magnitude or change in stance time variables were evident this interaction term was included in the final linear mixed model including all predictors (Additional file 2: Table S1). Footwear condition (defined as stability and neutral shoes) was entered as a fixed effect with independent predictors (i.e., change in lower limb kinematics, change in knee-GRF lever arm and change in resultant sagittal plane GRF magnitude) and any interactions terms as covariates in the model. The fixed effect estimates, 95% CI and *p* values were reported. All data were analysed using the SPSS (version 23, IBM) and *p* < 0.05 was used to indicate statistical significance.

## Results

Participant demographics are shown in Table 2. Included in the study were 29 pre-menarche girls, 20 eumenorrheic girls and 11 girls using the monophasic OCP. A mean value of 8.1 ± 5.1 pmol/L confirmed low estradiol levels at the time of testing (Table 2).

**Table 2:** Participant characteristics

Variable	Mean ± SD (*n* = 60)
Age (years)	15.6 ± 5.4
Weight (kg)	49.6 ± 13.8
Height (m)	1.6 ± 0.1
Estradiol (pmol/L)	8.1 ± 5.1
Thigh segment length (cm)	41.2 ± 3.5
Shank segment length (cm)	36.8 ± 3.2

*SD* standard deviation

### Differences in peak KFM, GRF and lower limb kinematics between footwear conditions

Analysis revealed no statistically significant differences in running velocity between footwear conditions (*p* > 0.05), yet a main effect of footwear was found for stance time (*p* < 0.001, Table 3). *Post-hoc* analysis revealed a longer stance time wearing stability (MD = 0.02, 95% CI 0.01, 0.02 s, *p* < 0.001) and neutral shoes (MD = 0.02, 95% CI 0.01, 0.02 s, *p* < 0.001) compared to barefoot, with no between shoe differences (*p* = 0.08, Table 3). A main effect of footwear for peak KFM was also found (*p* < 0.001, Table 3). The stability (MD = 0.42, 95% CI 0.36 to 0.49 Nm/kg, *p* < 0.001) and neutral (MD = 0.38, 95% CI 0.31 to 0.45 Nm/kg, *p* < 0.001) shoes resulted in a higher peak KFM during running compared to barefoot. No differences in the peak KFM between shoes were found (*p* = 0.06).

**Table 3:** Differences in biomechanical variables of interest between footwear and barefoot conditions. All variables are reported as mean ± standard deviation with the *P* value for between shoes and barefoot comparisons

Variable	Barefoot	Stability	Neutral	*P* value barefoot v high-support	*P* value barefoot v low-support	*P* value high-support v low-support
Peak KFM (Nm/kg)	2.24 ± 0.41	2.67 ± 0.41^*a*^	2.62 ± 0.39^*a*^	< 0.001	< 0.001	0.06
Running velocity (m/s)	3.12 ± 0.20	3.12 ± 0.21	3.14 ± 0.21	0.76	0.07	0.41
Stance time (s)	0.23 ± 0.03	0.25 ± 0.02^*a*^	0.25 ± 0.02^*a*^	< 0.001	< 0.001	0.08
Sagittal plane knee-GRF lever arm (mm)^c^	103.75 ± 18.05	119.50 ± 18.04^*ab*^	117.29 ± 17.05^*a*^	< 0.001	< 0.001	0.04
Sagittal plane resultant GRF magnitude (BW)^c^	2.48 ± 0.45	2.55 ± 0.44^*a*^	2.56 ± 0.44^*a*^	< 0.001	< 0.001	0.27
Hip flexion angle (°)^c^	37.64 ± 7.60	40.45 ± 8.04^*a*^	40.04 ± 7.58^*a*^	< 0.001	< 0.001	0.43
Knee flexion angle (°)^c^	46.96 ± 4.52	48.99 ± 4.84^*a*^	49.36 ± 4.10^*a*^	< 0.001	< 0.001	0.32
Knee flexion at initial contact (°)	19.11 ± 4.30	16.16 ± 4.40^*ab*^	17.64 ± 5.83^*a*^	< 0.001	0.02	0.04
Knee flexion excursion (°)	32.40 ± 4.79	36.21 ± 4.22^*a*^	36.31 ± 4.32^*a*^	< 0.001	< 0.001	0.77
Ankle dorsiflexion angle (°)^c^	19.01 ± 3.81	20.17 ± 3.08^*a*^	19.75 ± 3.37	0.01	0.12	0.12

*BW* bodyweight, *KFM* external knee flexion moment

^*a*^significantly different to barefoot

^*b*^significantly different to neutral

^c^at time of peak KFM

Similarly, main effects of footwear were found for all remaining variables outlined in Table 3. *Post-hoc* comparisons revealed a higher knee-GRF lever arm wearing stability shoes compared to neutral (MD = 2.23, 95% CI 0.11, 4.30 mm, *p* = 0.04) and barefoot conditions (MD = 15.75, 95% CI 13.41, 18.08 mm, *p* < 0.001). Furthermore, the neutral shoes increased the knee-GRF lever arm compared to barefoot (MD = 13.54, 95% CI 11.06, 16.02 mm, *p* < 0.001). Wearing both stability (MD = 0.07, 95% CI 0.03, 0.10 BW, *p* < 0.001), and neutral (MD = 0.08, 95% CI 0.05, 0.11, *p* < 0.001) shoes increased the sagittal plane resultant GRF magnitude compared to barefoot; however, no differences were found between shoe conditions (*p* > 0.05).

With respect to lower limb kinematics, the knee flexion angle at peak KFM in stability (MD = 2.03, 95% CI 1.16, 2.90°, *p* < 0.001) and neutral shoes (MD = 2.41, 95% CI 1.76, 3.04°, *p* < 0.001) was increased compared to barefoot, with no statistically significant between-shoe differences observed (*p* = 0.32). stability shoes significantly increased ankle dorsiflexion angle at peak KFM compared to barefoot (MD = 1.16, 95% CI 0.33, 2.00°, *p* = 0.01); however, no differences were found wearing neutral shoes compared to stability or barefoot conditions (*p* > 0.05). At the hip, the stability shoes (MD = 2.81, 95% CI 1.79, 3.82 °, *p* < 0.001) and neutral shoes (MD = 2.40, 95% CI 1.52, 3.28°, *p* < 0.001) increased the flexion angle at peak KFM compared to barefoot, with no between shoe differences evident (*p* = 0.43).

Differences between footwear and barefoot were also evident for knee excursion angle across the stance phase with higher values wearing stability (MD = 3.82, 95% CI 2.99, 4.65°, *p* < 0.001) and neutral shoes (MD = 3.92, 95% CI 3.19, 4.64°, *p* < 0.001) compared to barefoot. Surprisingly, knee flexion angle at initial contact was lower in stability shoes compared to neutral shoes (MD = 1.48, 95% CI 0.07, 2.90°, *p* = 0.04) and barefoot (MD = 2.95, 95% CI 1.96, 3.95°, *p* < 0.001), with the neutral shoes revealing lower angles at initial contact compared to barefoot (MD = 1.47, 95% CI 0.25, 2.70°, *p* = 0.02).

### Predictors underlying change in peak KFM between footwear and barefoot conditions

There was evidence of an interaction between footwear condition and the change in knee-GRF lever arm (*p*-value for interaction < 0.001, Additional file 2: Table S1). Subsequently, this interaction term was considered for each condition (barefoot and footwear) in the final regression model analysing factors associated with change in peak KFM between footwear and barefoot conditions. This model included a total of nine potential predictors (Table 4), of which, only the change in knee-GRF lever arm in barefoot (MD = 0.02, 95% CI 0.02, 0.03 mm, *p* < 0.001) and footwear conditions (MD = 0.02, 95% CI 0.01, 0.03 mm, *p* < 0.001) had a statistically significant association with a change in peak KFM. The change in sagittal-plane resultant GRF magnitude, footwear condition and change in hip, knee and ankle kinematics did not have statistically significant associations with a change in peak KFM in this regression model (*p* > 0.05, Table 4).

**Table 4:** Linear mixed model analysis for the change in peak KFM between footwear and barefoot conditions. Fixed effect estimates, 95% CI and *p* values are reported for each term analysed within the model

Predictors	Change in Peak KFM
	Fixed effect estimates, (95% CI), *p*-value
Footwear condition	0.04, (− 0.10, 0.18), *p* = 0.55
Change in sagittal plane knee-GRF lever arm (mm) in barefoot condition	0.02, (0.02, 0.03), *p* < 0.001
Change in sagittal plane knee-GRF lever arm in footwear condition	0.02, (0.01, 0.03), *p* < 0.001
Change in sagittal plane resultant GRF magnitude (BW)	−0.01, (− 0.06, 0.04), *p* = 0.72
Change in hip flexion angle (°)	− 0.004, (− 0.02, 0.01), *p* = 0.46
Change in knee flexion angle (°)	0.01, (− 0.001, 0.03), *p* = 0.07
Change in knee flexion at initial contact (°)	− 0.0003, (− 0.01, 0.01), *p* = 0.96
Change in knee flexion excursion (°)	0.003, (− 0.01, 0.03), *p* = 0.64
Change in ankle dorsiflexion angle (°)	− 0.0002, (− 0.01, 0.01), *p* = 0.97

*BW* bodyweight, *KFM* external knee flexion moment, *GRF* ground reaction force

## Discussion

Running is a popular form of exercise amongst adolescent girls and young women. This study found a higher peak KFM during running whilst wearing both stability and neutral shoes compared to barefoot, with no strong evidence of between-shoe differences in this sample. Furthermore, a novel finding of this study was a change in the knee-GRF lever arm is associated with a change in peak KFM wearing shoes compared to barefoot.

Higher running-related peak KFM in adolescent girls and young women wearing stability and neutral shoes compared to barefoot partly supports the primary hypotheses but does not support the hypothesis of a between-shoe difference in peak KFM. Previous studies have reported increased running-related peak KFM wearing stability shoes and neutral shoes compared to barefoot amongst mixed adult cohorts aged 26–29 years [8, 20], but between shoe comparisons have not been previously performed. The present study now extends these results to adolescent girls and young women and is the first to include a direct comparison of stability and neutral shoes. The lack of between-shoe differences in peak KFM suggests that the relevant shoe design features such as footwear pitch did not influence peak KFM.

The regression analysis revealed an association between a change in peak KFM and a change in the knee-GRF lever arm, rather than lower limb kinematics or the sagittal plane resultant GRF magnitude wearing shoes compared to barefoot. This novel finding suggests that future footwear modifications aiming to reduce peak KFM should consider shoe design features that have the potential to reduce the knee-GRF lever arm. Specifically, footwear pitch (i.e., the heel to toe offset, [17]) and midsole density/compliance may be important features contributing to difference in the knee-GRF lever arm.

Harder and/or thicker midsoles may contribute to alterations of the knee GRF lever arm wearing shoes compared to barefoot, as previous research indicates that thicker midsoles can reduce plantar sensation [30], and can lead to higher knee flexion kinetics and kinematics [21]. Although we did not measure midsole thickness, we recommend that future studies investigate the influence of midsole thickness on the knee-GRF lever arm in this population.

Footwear pitch may also have contributed to the knee-GRF lever arm findings, as both the stability and neutral shoes we tested possessed 13 mm and 10 mm heel to toe offsets respectively, compared to 0 mm while barefoot. Although no studies have examined the relationship between pitch and change in peak KFM or the knee-GRF lever arm, lowering the pitch of shoes may indeed influence these parameters by reducing ankle dorsiflexion and knee flexion angles towards barefoot levels. Support for this theory is provided by Lindenberg et al. (2011) who explored the association between heel height and knee flexion angle during a forward hopping task in collegiate females [22]. They reported that increasing heel raises from 0 mm to 24 mm, significantly increased the peak knee flexion angle. Moreover, a study by Chambon et al. (2015) found that wearing shoes with increasing pitch (0, 4 and 8 mm), reduced ankle dorsiflexion and increased knee flexion angle excursions compared to barefoot while running over ground [23], thereby indicating that footwear pitch may be a factor driving changes in peak KFM and/or the knee-GRF lever arm.

Contrary to these findings, a recent randomized controlled trial by Malisoux et al. (2017) reported the effect of 0, 6 and 10 mm pitch shoes on lower limb kinematics over a period of 6 months [31]. Surprisingly, there was no between-shoe differences for mid-stance knee flexion angle; however, the flexion angle decreased in all shoe conditions over the six-month period. It is important to note that participants in Malisoux et al. (2017) ran on an instrumented treadmill, which can produce opposite kinematic effects to over-ground running and, as such, may explain their contradictory findings [31]. In support, the aforementioned study by Chambon et al. (2015) also found that running surface (i.e., over-ground versus a treadmill) had the opposite effect regarding the pitch of shoes and knee kinematics [23]. Nonetheless, in the present study footwear pitch likely caused an increase in peak KFM via the knee-GRF lever arm, as our testing was performed over-ground.

Greater hip, knee and ankle flexion angles at time of peak KFM in shoes compared to barefoot were also found. Numerous other studies support these findings in both stability and/or neutral footwear [8, 20, 32, 33]. Although not relevant in explaining the increase in peak KFM with footwear, these kinematic alterations are still of relevance in the context of PFP given that recent systematic reviews report kinematic differences at the knee and ankle between individuals with and without PFP whilst wearing similar footwear styles [6, 34, 35]. Knowing that stability and neutral footwear generally increase knee and ankle kinematics associated with the development of PFP [6, 34, 35], shoes with a lower pitch (i.e., < 5 mm) may be beneficial in an adolescent cohort.

Although the mechanism by which footwear changed the knee-GRF lever arm was not explored, running-related spatiotemporal variables may be important to include in future studies. Specifically, examining the association between changes in stride length and knee-GRF lever arm distance between footwear conditions is suggested based on recent evidence demonstrating footwear-related effects on stride length and peak KFM [32, 36]. For example, Sinclair and colleagues (2016) revealed that stability shoes not only increased peak KFM, but also increased stride length in comparison to barefoot-inspired shoes [31]. While this suggests that stride length could indeed be related to the knee-GRF lever arm, which primarily dictates peak KFM, further investigation is required.

This study has a number of limitations. It included a healthy adolescent/young adult female cohort free of PFP; thus, no link can be made between footwear-related peak KFM and the risk of developing the condition. Further, prospective research is required to determine causality. In addition, only external moments and kinematic predictors of a change in peak KFM were included and there are likely other variables associated with a change in the peak KFM that were not explored in the present study. For instance, the lack of spatiotemporal variables may also differ between conditions and predict changes in peak KFM. As such, future studies should consider incorporating computational neuromusculoskeletal models to examine footwear-related changes in musculotendinous forces and internal joint loads [37]. As there is no gold standard method of characterizing stability and neutral footwear, this study utilised the footwear assessment tool to appraise footwear characteristics provided by the manufacturer (i.e., medial post) or subjectively assessed by the researchers (i.e., torsional, longitudinal and heel counter stiffness) [17]. Therefore, shoes used in the present study may not necessarily be classified as ‘high and ‘low’ support if alternative methods were used to characterize footwear type. Furthermore, only one particular brand of shoes was assessed and findings may not necessarily generalize to other brands of footwear.

## Conclusion

This study found evidence that running in commercially available stability and neutral shoes increased the peak KFM compared to barefoot in adolescent girls and young women. Contrary to our hypothesis, there were no difference in peak KFM between the two footwear types. A change in peak KFM was associated with a change in knee-GRF lever arm, but not to changes in the sagittal-plane resultant GRF magnitude or sagittal plane hip, knee or ankle kinematics wearing shoes compared to barefoot. Future studies should consider modifying footwear features to attenuate these higher knee loads in young females given that higher peak KFM may be associated with a greater risk of developing pathological conditions such as PFP.

## Additional files

Additional file 1: **Figure S1.** Technical features of the stability and neutral support shoes. The stability shoes (ASICS Kayano-GS, A) featured a (i) heel stack height= 25mm, (ii) forefoot stack height= 12mm, (iii) footwear pitch= 13mm and (iv) shoe mass= 260g. In contrast, the neutral shoes (ASICS Zaraca 3, B) featured a (i) heel stack height= 28mm, (ii) forefoot stack height= 18mm, (iii) footwear pitch= 10mm and (iv) shoe mass= 240g. (DOCX 19 kb)

Additional file 2: **Table S1.** Interactions between footwear condition and predictors from linear mixed models. Results depict the preliminary step testing for interactions between footwear condition and biomechanical predictors for the change in peak KFM (dependant variable). Interactions with *p*-values < 0.05 were included in the final mixed model (Table 4). Fixed effect estimates, 95% CI and *p* values are reported for each term analysed within the model. (DOCX 7306 kb)

## Abbreviations

ANOVA: Analysis of variance
CI: Confidence interval
FAT: Footwear assessment tool
GRF: Ground reaction force
HEAG: Human ethics advisory group
HESC: Human ethics sub committee
KFM: Knee flexion moment
LPI: Lateral preference inventory
LSD: Least square’s difference, inventory
MD: Mean difference
OCP: Oral contraceptive pill
PFP: Patellofemoral pain
