# Supplementary material for: Differences and mechanisms underpinning a change in the knee flexion moment while running in stability and neutral footwear among young females
Source: J Foot Ankle Res. 2019 Jan 8;12:1. doi: 10.1186/s13047-018-0307-9 (PMC6323812; doi:10.1186/s13047-018-0307-9)
Supplement: Supplementary file 1 — Figure S1. Technical features of the stability and neutral support shoes. The stability shoes (ASICS Kayano-GS, A) featured a (i) heel stack height= 25mm, (ii) forefoot stack height= 12mm, (iii) footwear pitch= 13mm and (iv) shoe mass= 260g. In contrast, the neutral shoes (ASICS Zaraca 3, B) featured a (i) heel stack height= 28mm, (ii) forefoot stack height= 18mm, (iii) footwear pitch= 10mm and (iv) shoe mass= 240g. (DOCX 19 kb) [file 13047_2018_307_MOESM1_ESM.docx]

B

A

**Figure S1** Technical features of the stability and neutral support shoes. The stability shoes (ASICS Kayano-GS, A) featured a (i) heel stack height= 25mm, (ii) forefoot stack height= 12mm, (iii) footwear pitch= 13mm and (iv) shoe mass= 260g. In contrast, the neutral shoes (ASICS Zaraca 3, B) featured a (i) heel stack height= 28mm, (ii) forefoot stack height= 18mm, (iii) footwear pitch= 10mm and (iv) shoe mass= 240g.
