# Supplementary material for: Differences and mechanisms underpinning a change in the knee flexion moment while running in stability and neutral footwear among young females
Source: J Foot Ankle Res. 2019 Jan 8;12:1. doi: 10.1186/s13047-018-0307-9 (PMC6323812; doi:10.1186/s13047-018-0307-9)
Supplement: Supplementary file 2 — Table S1. Interactions between footwear condition and predictors from linear mixed models. Results depict the preliminary step testing for interactions between footwear condition and biomechanical predictors for the change in peak KFM (dependant variable). Interactions with p-values < 0.05 were included in the final mixed model (Table 4). Fixed effect estimates, 95% CI and p values are reported for each term analysed within the model. (DOCX 7306 kb) [file 13047_2018_307_MOESM2_ESM.docx]

**Table S1.** Interactions between footwear condition and predictors from linear mixed models. Results depict the preliminary step testing for interactions between footwear condition and biomechanical predictors for the change in peak KFM (dependant variable). Interactions with p-values < 0.05 were included in the final mixed model (Table 4). Fixed effect estimates, 95% CI and *p* values are reported for each term analysed within the model.

| Predictors | Change in Peak KFM  Fixed effect estimates, (95% CI), *p*-value |
| --- | --- |
| Stability ×change in sagittal plane knee-GRF lever arm (mm) | 0.02, (0.01, 0.03), *p*= 0<0.001 |
| Neutral ×change in sagittal plane GRF lever arm (mm) | 0.02, (0.02, 0.03), *p*=0<0.001 |
| Stability ×change in sagittal plane resultant GRF magnitude (BW) | -0.02, (-0.05, 0.05), *p*=0.96 |
| Neutral ×change in sagittal plane resultant GRF magnitude (BW) | 0.29, (-0.27, 0.82), *p*=0.32 |
| Stability ×change in knee flexion angle (°) | 0.01, (-0.01, 0.03), *p*=0.31 |
| Neutral ×change in knee flexion angle (°) | 0.02, (-0.01, 0.04), *p*=0.19 |
| Stability ×change in knee flexion excursion angle (°) | 0.01, (-0.01, 0.04), *p*=0.12 |
| Neutral ×change in knee flexion excursion (°) | -0.01, (-0.03, 0.02), *p*=0.73 |
| Stability ×change in knee flexion at initial contact (°) | 0.001, (-0.01, 0.02), *p*=0.66 |
| Neutral ×change in knee flexion at initial contact (°) | -0.002, (-0.02, 0.02), *p*=0.81 |
| Stability ×change in ankle dorsiflexion angle (°) | -0.002, (-0.02, 0.01), *p*=0.71 |
| Neutral ×change in ankle dorsiflexion angle (°) | 0.002, (-0.01, 0.02), *p*=0.85 |
| Stability ×change in hip flexion angle (°) | -0.005, (-0.02, 0.01), *p*=0.38 |
| Neutral ×change in hip flexion angle (°) | -0.003, (-0.02, 0.01), *p*=0.64 |
| Stability ×change in stance time (s) | -3.95, (-8.55, 0.65), *p*=0.09 |
| Neutral ×change in stance time (s) | -1.66, (-6.40, 3.06), *p*=0.49 |

BW= bodyweight, KFM= external knee flexion moment, GRF= ground reaction force
